# High-Glucose-Induced Endothelial Cell Injury Is Inhibited by a Peptide Derived from Human Apolipoprotein E

**DOI:** 10.1371/journal.pone.0052152

**Published:** 2012-12-19

**Authors:** Partha S. Bhattacharjee, Tashfin S. Huq, Valencia Potter, Anna Young, Ian R. Davenport, Richard Graves, Tarun K. Mandal, Christian Clement, Harris E. McFerrin, Syed Muniruzzaman, Shubha K. Ireland, James M. Hill

**Affiliations:** 1 Department of Biology, Xavier University of Louisiana, New Orleans, Louisiana, United States of America; 2 Department of Ophthalmology, Louisiana State University Health Sciences Center, New Orleans, Louisiana, United States of America; 3 Tulane School of Medicine, New Orleans, Louisiana, United States of America; 4 University of South Alabama School of Medicine, Mobile, Alabama, United States of America; 5 Allegheny College, Meadville, Pennsylvania, United States of America; 6 College of Pharmacy, Xavier University of Louisiana, New Orleans, Louisiana, United States of America; 7 Neuroscience Center, Louisiana State University Health Sciences Center, New Orleans, Louisiana, United States of America; 8 Department of Microbiology, Immunology and Parasitology, Louisiana State University Health Sciences Center, New Orleans, Louisiana, United States of America; 9 Department of Pharmacology, Louisiana State University Health Sciences Center, New Orleans, Louisiana, United States of America; UAE University, United Arab Emirates

## Abstract

Although the importance of human apolipoprotein E (apoE) in vascular diseases has clearly been established, most of the research on apoE has focused on its role in cholesterol metabolism. In view of the observation that apoE and its functional domains impact extracellular matrix (ECM) remodeling, we hypothesized that apoE could also confer protection against ECM degradation by mechanisms independent of its role in cholesterol and lipoprotein transport. The ECM degrading enzyme, heparanase, is secreted by cells as pro-heparanase that is internalized through low-density lipoprotein (LDL) receptor-related protein-1 (LRP-1) to become enzymatically active. Both apoE and pro-heparanase bind the LRP-1. We further hypothesized that an apoE mimetic peptide (apoEdp) would inhibit the production of active heparanase by blocking LRP-1-mediated uptake of pro-heparanase and thereby decrease degradation of the ECM. To test this hypothesis, we induced the expression of heparanase by incubating human retinal endothelial cells (hRECs) with high glucose (30 mM) for 72 hours. We found that elevated expression of heparanase by high glucose was associated with increased shedding of heparan sulfate (ΔHS) and the tight junction protein occludin. Treatment of hRECs with 100 µM apoEdp in the presence of high glucose significantly reduced the expression of heparanase, shedding of ΔHS, and loss of occludin as detected by Western blot analysis. Either eye drop treatment of 1% apoEdp topically 4 times a day for 14 consecutive days or intraperitoneal injection (40 mg/kg) of apoEdp daily for 14 consecutive days in an *in vivo* mouse model of streptozotocin-induced diabetes inhibited the loss of tight junction proteins occludin and zona occludin- 1 (ZO-1). These findings imply a functional relationship between apoE and endothelial cell matrix because the deregulation of these molecules can be inhibited by a short peptide derived from the receptor-binding region of apoE. Thus, strategies targeting ECM-degrading enzymes could be therapeutically beneficial for treating diabetic retinopathy.

## Introduction

Diabetes mellitus is associated with both macro- and microangiopathy [1], [2]. Diabetic microangiopathy is characterized by retinopathy and nephropathy [3], [4]. In hyperglycemia, the first targets are endothelial cells (ECs) of blood vessels. ECs produce heparan sulfate proteoglycan (HSPG), which is an important constituent of the extracellular matrix (ECM) [5], [6]. ECM is important for maintaining vascular integrity and function as depot of several angiogenic (blood vessel growth) growth factors and inflammatory cytokines [7], [8]. ECMs present in the plasma membrane and basement membrane of ECs function as a cementing substance, and being negatively charged, repel plasma proteins; ECM degradation leads to increased vascular permeability and incites angiogenesis and inflammation through release of growth factors and inflammatory molecules [9]. HSPG contains a sugar chain, heparan sulfate (HS), linked to a protein core called proteoglycan (PG). The shedding of HS from HSPG is an important hallmark of hyperglycemic injury to ECs correlated to diabetic retinopathy and nephropathy [10]–[12].

ECs lining the inner wall of blood vessels are held together by tight junction proteins, including occludin and zona occludin-1 (ZO-1) which bind to intracellular actin and create the tight seal of the blood-retinal barrier (BRB) [13]. Degradation of tight junctions in ECs leads to leaky vessels and fluid exudation in the neighboring area leading to development of diabetic retinopathy and macular edema [14]. Leaky blood vessels are associated with deposition of lipoprotein exudates called drusen, which eventually results in proliferative retinopathy and microaneaurysms leading to vision loss [15].

Hyperglycemia is known to induce the expression of the ECM-degrading enzyme, heparanase. Heparanase degrades HSPG leading to the loss of HS [16]. Understanding how heparanase works may lead to a valuable medical breakthrough related to diabetes, angiogenesis, cancer, and many inflammatory conditions. Cells initially produce heparanase in their inactive form, called pro-heparanase (65 kDa) and secrete it to the extracellular fluid. This inactive pro-heparanase needs to bind to low-density lipoprotein (LDL) receptor-related protein-1 (LRP-1) present in the HSPG component of ECM to be internalized. Following uptake/internalization, pro-heparanase is proteolytically cleaved yielding ∼8 and ∼50 kDa proteins that heterodimerize to form active heparanase and remain localized [17].

Human apolipoprotein E (apoE) primarily functions as a cholesterol transporter and also uses LRP-1 for internalization into the cell [18]. We focused on the receptor-binding region located between amino acids residues 142–147 which are responsible for apoE binding to LRP-1 present in the ECM [18]. See Table 1 for the structure and total positive charges on the apoE dipeptide. Compared to the holoprotein apoE, short peptides derived from this receptor-binding region are reported to cross the BRB [19]. Unlike the short monomer peptide derived from this region, a tandem repeat dimer peptide had been reported by us and others to be antimicrobial, anti-angiogenic and anti-tumorigenic [20]–[23]. The tandem repeat design of the peptide was adopted to create the α-helical structure formed in the N-terminal apoE (130–164) under hydrophobic conditions [22]. We have shown that the ApoE dipeptide has anti-neovascularization and anti-inflammatory properties [23]. This has been shown by systemic and topical administration of the apoE dipeptide. Our hypothesis is that the apoE dipeptide will act in the mouse retina to inhibit endothelial cell injury similar to the way that this mimetic peptide has shown potency in the rabbit eye model and in the mouse tumorigenic model. Our rationale was that a peptide mimicking the LRP binding domain of human apoE would competitively inhibit the production of enzymatically active heparanase by hRECs and would result in inhibition of BRB breakdown.

**Table 1:** Comparative physical properties of two natural cationic peptides.

Peptide	Arginines	Lysines	Total residues	Sequence	Total positive charges
TAT(48–60)	**6**	**2**	**9**	**GRKKRRQRRRPPQ**	**+8**
**ApoEdp**	**6**	**4**	**18**	**LRKLRKRLLLRKLRKRLL**	**+10**

## Results

### ApoEdp inhibits the high glucose-induced expression of active heparanase (50 kDa) in human retinal endothelial cells (hRECs)

**Figure 1:**
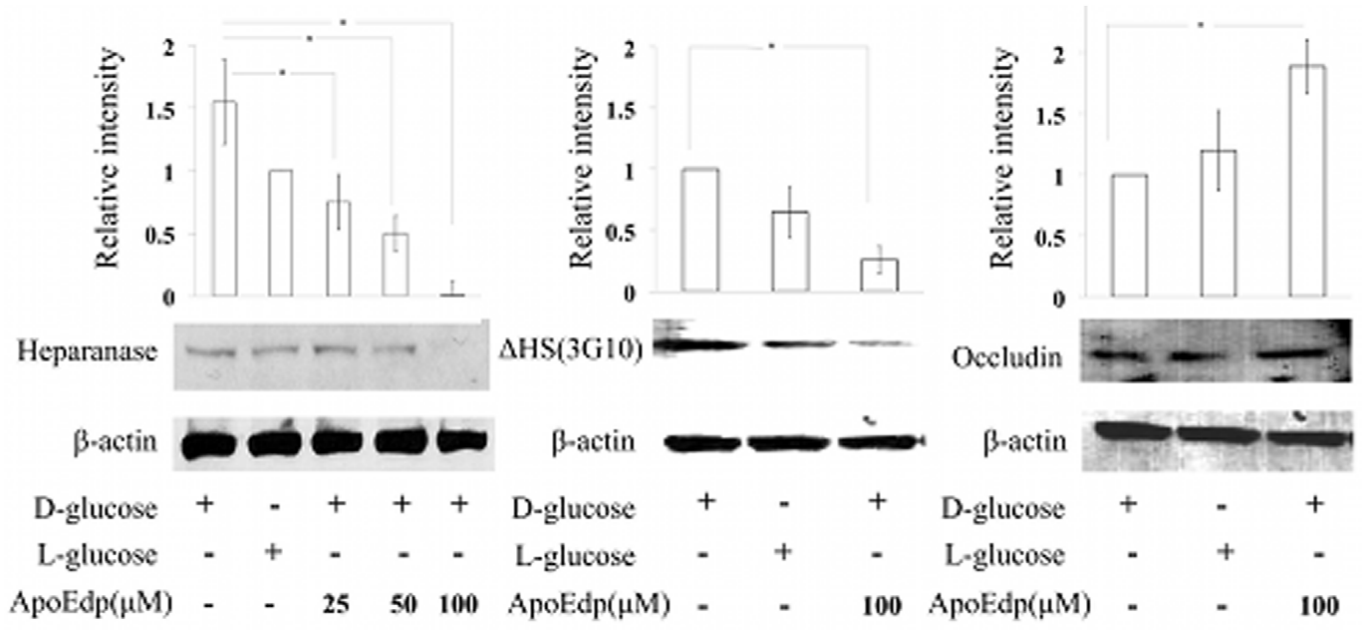
The protective effect of apoEdp on hRECs injured by high glucose in the cell medium. (**A**). The hRECs were treated with varying doses of 25, 50, and 100 µM of apoEdp in the presence of 30 mM D-glucose or L-glucose (osmotic control). Western blot analysis of proteins extracted from high sugar-injured cells following 72 hours of apoEdp treatment revealed dose-dependent inhibition of heparanase expression. The 100 µM apoEdp treatment inhibited (*P≤0.05) heparanase expression to the lowest level of the 3 treatment groups assessed. (**B**). To detect the effect of hyperglycemia-induced HS loss (Δ-HS) in hRECs treated with 100 µM of apoEdp, a commercial monoclonal antibody (3G10) specific to neoepitope generated by heparanase digestion of HSPG was used. A 100 µM apoEdp treatment significantly inhibited (*P≤0.05) the loss of Δ-HS in hRECs after 72 hours of high glucose treatment compared to mock treatment (high glucose but no apoEdp treatment). (**C**). ApoEdp (100 µM) treatment prevented the loss of tight junction protein occludin in hRECs under hyperglycemic conditions. The hRECs were incubated for 72 hours with or without apoEdp (100 µM) in the presence of hyperglycemia and assayed for the expression of occludin. Each value represents the mean ± SE of results in three independent experiments (*P≤0.05). β-actin served as the loading control.

To determine the protective effect of apoEdp against high sugar (30 mM)-induced injury to hRECs, cells were treated with different doses (25, 50 and 100 µM) of apoEdp for 72 hours. The sugar and apoEdp treatment was continued every day, and after 72 hours, cells were harvested to extract total protein and processed for Western blot analysis. The primary antibody used was specific for the detection of the active form (50 kDa) of heparanase. As shown in Figure 1A, a significant dose-dependent inhibition of heparanase expression was observed with nearly complete inhibition at 100 µM concentration of apoEdp (*P*≤0.05).

### ApoEdp prevents the high glucose-induced loss of heparan sulfate (Δ-HS) in hRECs

To investigate the high sugar effect on hREC surface HSPG, we used a commercial monoclonal antibody specific for Δ-HS (3G10), which is a neo-epitope that includes the Δ-glucuronate generated by heparanase-mediated digestion of heparan sulfate. As shown in Figure 1B, 100 µM of apoEdp treatment significantly (*P*≤0.05) attenuated the loss of Δ-HS compared to mock treatment (no apoEdp), in the presence of high sugar (30 mM) for 72 hours.

### ApoEdp prevents the high glucose-induced loss of tight junction protein occludin in hRECs

To determine the protective effect of apoEdp on the high sugar-induced loss of occludin in hRECs, we assessed the expression of occludin in hRECs treated with high sugar (D-glucose or L-glucose) with or without 100 µM apoEdp for 72 hours. The level of occludin expression was measured following 72 hours of high sugar and apoEdp treatment. As shown in Fig. 1C, apoEdp treatment significantly inhibited the loss of occludin in D-glucose-treated hRECs (*P*≤0.05).

### ApoEdp treatment inhibits the retinal tight junction protein expression in streptozotocin-induced diabetic (hyperglycemic) mice

**Figure 2:**
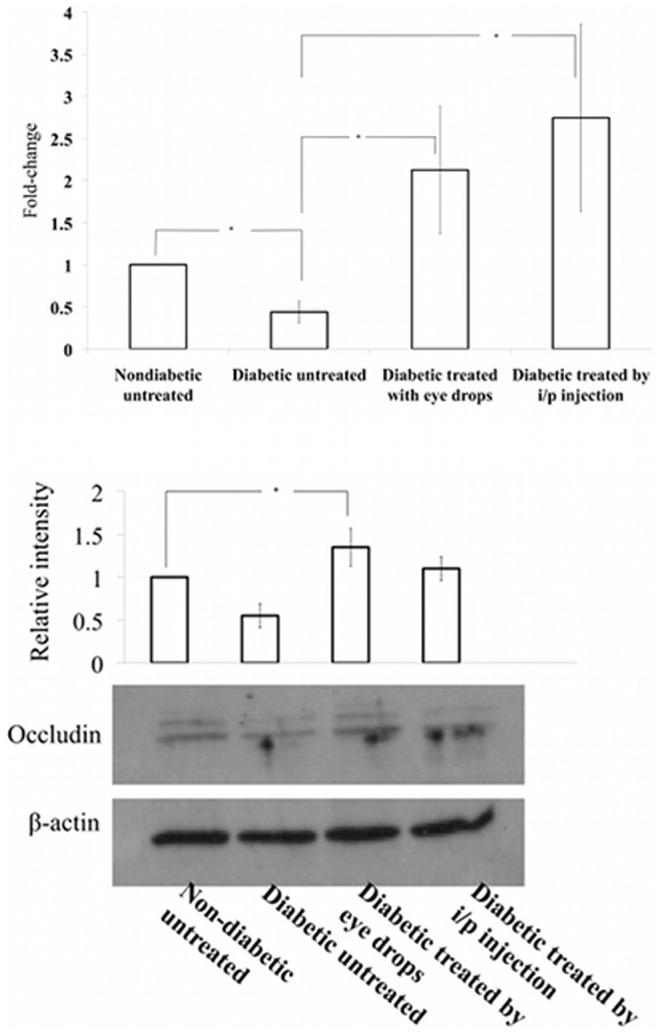
ApoEdp treatment prevents retinal tight junction protein loss diabetic mice. (**A**). RNA samples isolated from different experimental groups of mice retinas were assessed for occludin-specific mRNA using one-step real-time RT-PCR. ApoEdp treatment increased retinal occludin specific mRNA>2-fold in drop-treated eyes and >2.5 fold in animals given intraperitoneal injection. (**B**). ApoEdp treatment inhibits the loss of retinal occludin-specific protein expression in streptozotocin-induced diabetic mice as determined by Western blot analysis. Significantly upregulated expression of occludin was detected in both routes (topical and intraperitoneal) in the retinas of diabetic mice.

To investigate the effect of eye drops (1% apoEdp) given topically for 14 consecutive days or systemic intraperitoneal injection (40 mg/kg) for 14 consecutive days of apoEdp on the retinal tight junction protein occludin, mice were euthanized, the eyes were enucleated, and retinas were dissected. The dissected retinas were processed to harvest total RNA and one-step real-time RT-PCR assays were performed using mouse occludin-specific mRNA primer/probes. As shown in Figure 2A, both topical administration and intraperitoneal injection of apoEdp significantly inhibited the down-regulation of occludin-specific mRNA compared to diabetic non-treated mouse retinas. To correlate the occludin-specific mRNA data at protein level, total proteins extracted from retinas of different experimental groups (Figure 2B) were analyzed using Western-blot to detect occludin protein expression. Both eye drops and intraperitoneal injection of apoEdp significantly attenuated the loss of occludin in diabetic mice (Figure 2B).

### ApoEdp treatment restores ZO-1 protein expression in streptozotocin-induced diabetic mice retinas as determined by immunohistochemistry

**Figure 3:**
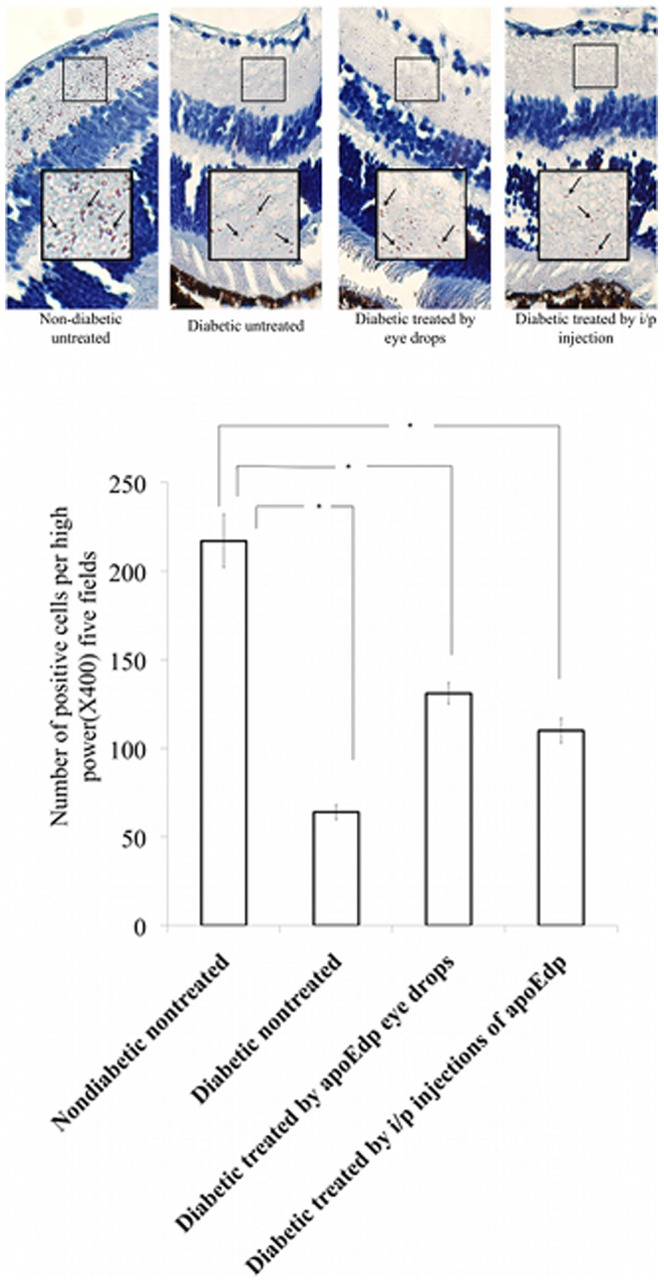
ApoEdp treatment restores retinal ZO-1 protein in diabetic mice as determined by immunohistochemistry. (**A**). Immunohistochemical analysis of formalin-fixed sections of mouse retinas were stained with zona occludin-1(ZO-1) specific antibody. Mean ±SEM of ZO-1 positive cells in five high power (×400) fields were counted. The arrows in the four photos indicate a ZO-1 positive cell. (**B**): The immunohistochemical quantitative analysis of ZO-1 protein in mouse retinas. Four treatment groups were used. The number of positive cells per high-power field is determined and the (mean ±SEM) of the ZO-1 positive cells are provided. The significant difference was detected in the ZO-1 cells between the diabetic, untreated eye, and the two groups of apoEdp treated mice retinas. Treatment with either topical 1% apoEdp drops or intraperitoneal injections of apoEdp for 14 consecutive days significantly improved the relationship of the number of increased positive cells compared to the untreated, diabetic retina.

To investigate the effect of apoEdp on attenuation of cell-to-cell adhesion-type tight junction protein zona occludin 1 (ZO-1), immunohistochemical detection of ZO-1 was performed in formalin-fixed deparafinized sections of mouse retinas of different experimental groups. Cells expressing ZO-1 were identified by the arrows in the photographs (Figure 3A). Figure 3B is the quantitative immunohistochemical analysis of ZO-1 protein in mouse retinas. The number of positive cells was determined in randomly chosen fields of view at high power (×400) for the retinas. The untreated diabetic mice had significantly fewer positive cells (p<0.05) than that of the non-diabetic, untreated (normal) control. Treatment with either topical 1% apoEdp drops or intraperitoneal injections of apoEdp for 14 consecutive days significantly increased the number of ZO-1 positive cells compared to the untreated diabetic retina.

### The apoEdp is a corneal penetrating peptide

The apoEdp peptide (LRKLRKRLLLRKLRKRLL) is rich in cationic amino acids lysine and arginine. ApoEdp is more cationic than HIV-1 Tat cell penetrating peptide (Table 1). We hypothesized that apoEdp could act as a corneal penetrating peptide. To prove our hypothesis, two corneal permeability studies of apoEdp were performed in mouse eyes. Our results (Table 2) suggest that apoEdp is a corneal penetrating peptide and applied topically, it reaches the anterior chamber of the eye within 3 hours. We suggest that apoEdp may be used for non-invasive treatment of posterior eye diseases. Results from these analyses are shown in Table 2.

**Table 2:** ApoEdp is a corneal penetrating peptide.

Sample	Measure concentration (µg/ml)	Total (ng)
0 hour, left eye	*	*
1 hour, left eye	*	*
2 hours, left eye	0.004	3.89
3 hours, left eye	0.058	58.08
0 hours, right eye	*	*
1 hour, right eye	0.022	2.22
2 hours, right eye	0.006	5.9
3 hours, right eye	0.036	36.3

* **Non detectable**.

### ApoEdp inhibits the up-regulated expression of VEGF in the retinas of diabetic mice

**Figure 4:**
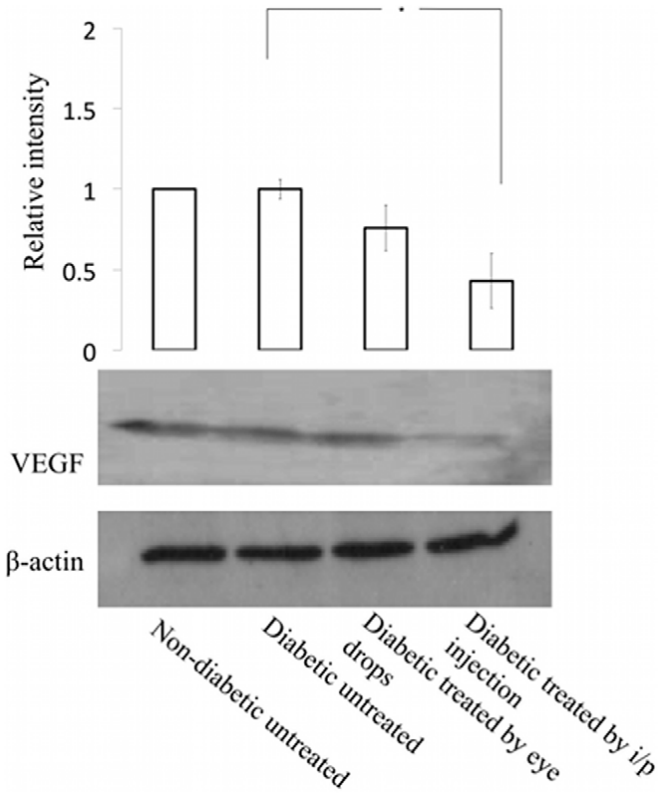
ApoEdp inhibits VEGF in the retinas of diabetic mice. Total protein extracts from the mouse retinas of diabetic mice were analyzed by Western blot to detect the VEGF expression. ApoEdp treatment significantly inhibited the expression of VEGF in diabetic mice retinas.

Upregulation of VEGF expression is reported as one of the reasons for the loss of tight junction proteins in diabetic mice [24]. Western blot analysis of total proteins extracted from diabetic mouse retinas revealed significant inhibition of VEGF in the retinas of apoEdp-treated diabetic mice compared to untreated diabetic mice (Figure 4).

## Discussion

Our study is the first to report that topical eye drop treatment of apoE mimetic peptide (apoEdp) is an effective and non-invasive approach to treat diabetes-induced retinal EC injury. We report that treatment with apoEdp reversed increased expression of heparanase, known to play a role in BRB breakdown. Breakdown of the BRB leads to diabetic retinopathy; subsequent development of macular edema is a major cause of visual loss in diabetic patients [25], [26]. Our study demonstrates that either 14-day eye drop application or systemic intraperitoneal injections of apoEdp reversed increased expression of heparanase and associated loss of HS and tight junction protein occludin and ZO-1.

Increased vascular permeability in DR is reported to result in a concomitant increase of VEGF. The VEGF pathway is known to play a role in breakdown of the BRB through a mechanism involving the down regulation of tight junction proteins in the retinal microvasculature [24].

Our results suggest that even via topical application of eye drops, apoEdp reached the mouse aqueous chamber through the cornea. This is not the first use of apoEdp as an ocular treatment. Previously, we reported that topical eye drop application of apoEdp inhibits VEGF-induced corneal angiogenesis and herpes virus-induced corneal neovascularization in mouse and rabbit eyes [20], [21], [23].

**Figure 5:**
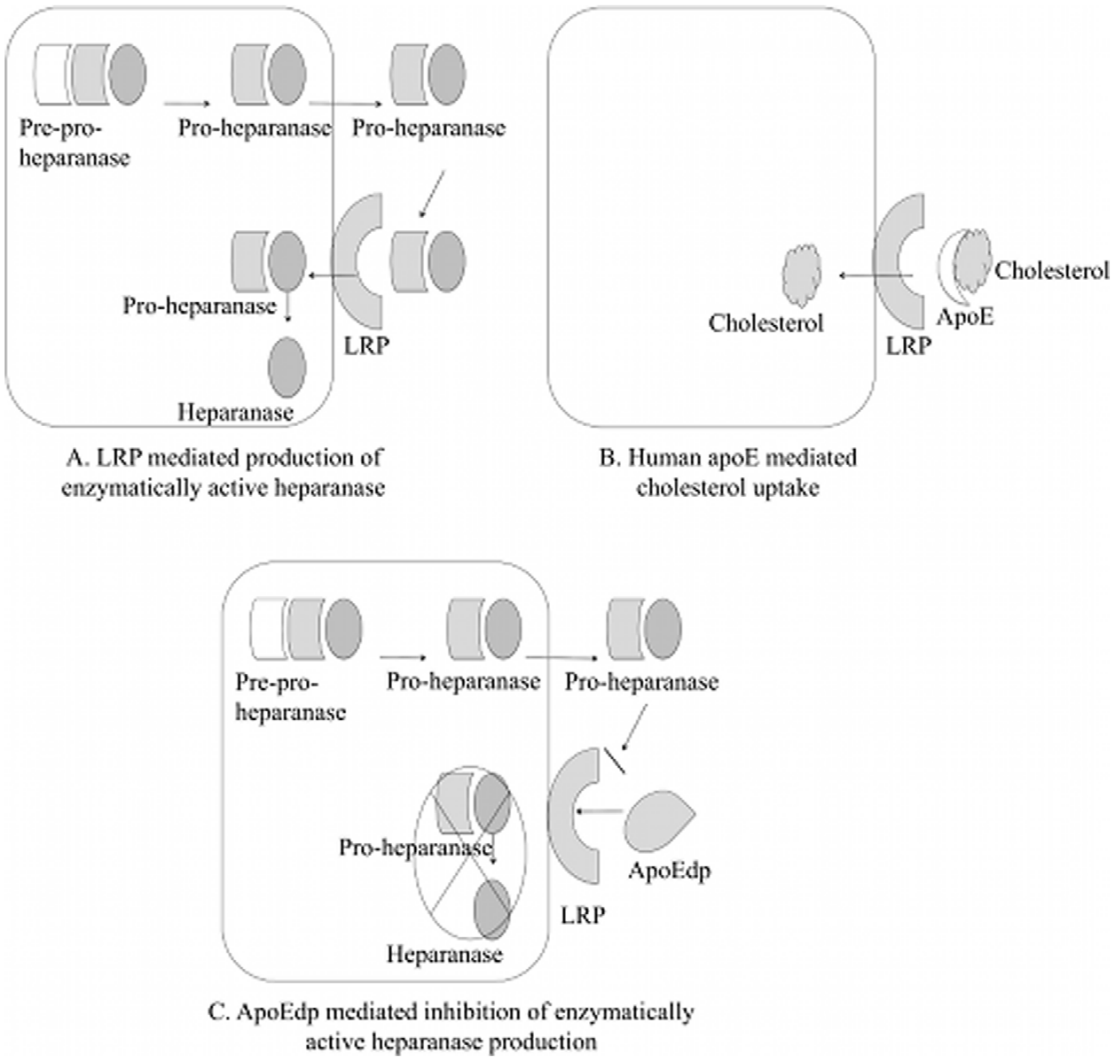
Possible mechanisms of apoEdp mediated heparanase inhibition. (A). Heparanase is produced as a larger precursor protein (pro-heparanase). LRP-1 on ECs known to rapidly bind the secreted pro-heparanase and transfer the internalized pro-heparanase to late endosomes/lysosomes. Pro-heparanase is proteolytically cleaved into enzymatically active form in intracellular lysosomal/endosomal compartments and remains localized. (B). Human apoE protein binds to the same receptor LRP present on the cell surface ECM. (C) The apoEdp competitively inhibits hREC uptake and internalization through LRP-1 arresting the production of active heparanase.

The exact mechanism of apoEdp function in preventing EC injury is still unknown. However, we have hypothesized that apoEdp competitively inhibits the EC uptake and internalization of pro-heparanase through LRP-1, arresting the production of active heparanase. Heparanase is produced as a larger precursor protein (pro-heparanase). LRP-1 present on the surface of ECs are known to rapidly bind secreted pro-heparanase and transfer the internalized pro-heparanase to late endosomes/lysosomes. Pro-heparanase is proteolytically cleaved into its enzymatically active form in the intracellular lysosomal/endosomal compartments and remains localized. The binding of pro-heparanase is mediated by cell surface HSPG, LRP-1, and mannose-6-phosphate receptors [17]. Since both apoE and heparanase use the same LRP-1 receptor present on the cell surface ECM, we rationalized (Figure 5) that competitive inhibition between precursor heparanase and apoEdp (derived from the LRP receptor binding region of apoE) would prevent active heparanase formation needed for ECM degradation and hence can alleviate or even prevent diabetic retinopathy. Further experiments are required to determine whether this is the case.

We have demonstrated that hyperglycemia-induced up-regulated expression of active heparanase (50 kDa) in ECs was inhibited by apoEdp treatment *in vitro*. Thus, therapeutic inhibition and prevention of microvascular diseases associated with diabetes could be addressed using apoEdp to inhibit heparanase activation.

Furthermore, EC injury induced by hyperglycemia results in HSPG degradation, which is characterized by HS (Δ HS) shedding, resulting in dysregulation of HSPG anionic barrier and subsequent increase in microvessel permeability. HS degradation is reported to contribute to diabetic vascular complications [27]. Our studies suggest that apoEdp prevents the shedding of HS (Δ HS) associated with high glucose-induced up regulated expression of heparanase.

**Figure 6:**
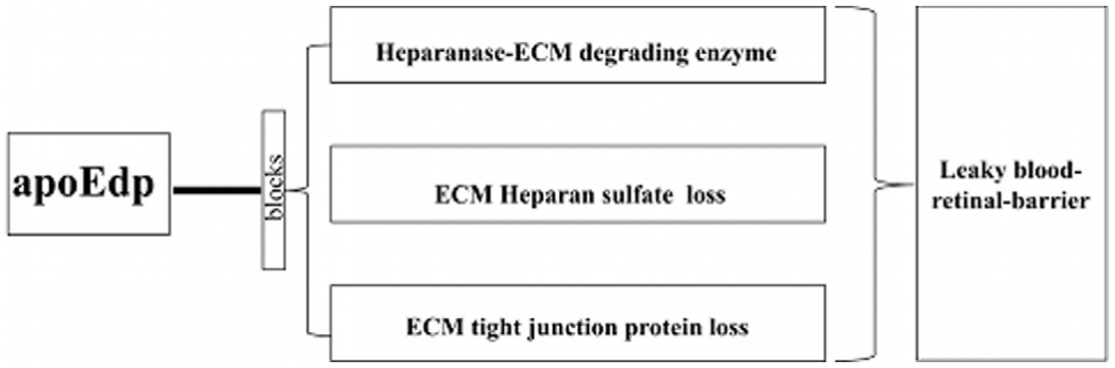
ApoEdp inhibits diabetic endothelial cell injury: Working model of apoEdp-mediated inhibition of hyperglycemia-induced expression of active heparanase by hRECs and related degradation of ECM.

We also found that high glucose-induced up-regulated expression of heparanase by ECs was accompanied by a loss of tight junction proteins *in vitro* and *in vivo*. Occludin and zona occludin (ZO-1) are well-characterized tight junction proteins of the BRB [13]. We targeted occludin and ZO-1 since their loss is known to be directly related to BRB permeability [13]. Based on the available evidence, we suggest that apoEdp could attenuate hypergycemia-induced BRB breakdown (Figure 6).

Cell surface HSPGs are reported to work as receptor sites for many natural and synthetic cell penetrating peptides (CPPs) [28]. The most studied CPP is HIV-1 derived TAT (48–60), which is rich in the positively charged amino acid arginine [29]. Cationic peptides rich in arginine (R) and lysine (K) were reported to play a crucial role in cell membrane penetration and internalization [30]. Once the positive charges are neutralized by the negatively charged HSPG, the less polar ion complex can cross the cell membrane [28]. The ApoEdp used in this study is even richer in positively charged amino acids arginine and lysine and is assumed to have more cell penetrating ability compared to TAT (Table 1). Secondary amphipathicity resulting from the α-helix conformation of cationic peptides has been reported to enhance cellular uptake [31]. As shown in Table 2, a single application of 1% eye drop treatment resulted in a measurable amount of apoEdp in the mouse aqueous humor. However, the therapeutic value of the concentration of apoEdp in aqueous humor reached after 3 hours is unknown. Current studies are underway to explore the ability of apoEdp to penetrate into the posterior chamber of the eye in a rabbit model.

In summary, our studies suggest an innovative approach of non-invasive therapeutic strategies for treating diabetic vascular complications.

## Methods

### Ethics Statement

The Xavier University of Louisiana Institutional Animal Care and Use Committee approved this study (protocol 02-182009-1B). All studies were performed in accordance with the Institute of Laboratory Animal Research (NIH, Bethesda, MD) Guide for the Care and Use of Laboratory Animals.

### Cells and peptide

Primary cultures of human retinal microvascular endothelial cells (hRECs) were purchased from the Applied Cell Biology Research Institute (Kirkland, WA) and grown on attachment factor-coated plates in Cell Systems Complete (CSC) medium (Cell Systems, Kirkland, WA) supplemented with 20% fetal bovine serum, culture boost (animal-derived growth factors), and Bac-Off (antibiotic, Cell Systems). The primary hRECs used were in between passages 4 and 6. The apoE mimetic peptide (apoEdp) was synthesized (Genemed, Arlington, TX) with a purity of greater than 95%. The 18 amino acid (Ac-LRKLRKRLLLRKLRKRLL-amide) tandem-repeat dimer peptide (apoEdp) was derived from the human apolipoprotein E receptor-binding region between residues 141 and 149, as described previously [22], [23]. ApoEdp treatment was performed in cell cultures containing high (30 mM) D-glucose or L-glucose (Sigma Aldrich, St. Louis, MO).

### Western Blotting

The hRECs were treated for 72 hours with/without apoEdp in the presence of high D- or L-glucose and were harvested in M-per containing protease inhibitor cocktail. The protein concentration was determined by using a BCA protein assay kit (Pierce, Rockford, IL). Equal amounts of protein were separated by electrophoresis on 5–20% sodium dodecyl sulfate-polyacrylamide gel electrophoresis and were transferred electrophoretically onto nitrocellulose membranes (Amersham, Little Chalfont, UK). The membranes were blocked for 1 hour in 5% nonfat milk. After blocking, the membranes were incubated overnight with anti-HPA-1 (human heparanase; 1∶1000, Santa Cruz Biotechnology, Santa Cruz, CA), anti-occludin (1∶1000; Santa Cruz Biotechnology), anti-mVEGF (1∶1000; Santa Cruz Biotechnology), and anti-ΔHS (3G10; 1∶ 1000, Santa Cruz Biotechnology) at 4°C. After being washed with PBS-T, the membranes were incubated for 1 hour at room temperature with horseradish peroxidase-conjugated anti-rabbit IgG or anti-mouse IgG (1∶10,000, Pierce) in PBS-T and 1% nonfat milk. To ensure the equal loading of protein in each lane, the blots were stripped and re-probed with an antibody against β-actin. The relative intensity values were normalized to control values. In all in vitro cell culture studies, we considered L-glucose treatment(no apoEdp) as isotonic control and all Western blot analysis was set for 1.0. In all in vivo mouse studies, the non-diabetic was considered as control and the Western blot analysis is set to 1.0.After scanning the blots using a flatbed scanner, the band intensities were analyzed using the ImageJ (NIH).

### Extraction of RNA, reverse transcription, and PCR

Following euthanasia, mouse eyes were enucleated and placed immediately in RNA-Later (Qiagen, Santa Clara, CA). Total cellular RNA was isolated using the RNeasy Mini Kit as specified by the manufacturer (Qiagen). Gene expression at RNA level per 100 ng of total RNA was measured using a one-step RT-PCR kit (Bio-Rad, Hercules, CA). The occludin gene was analyzed to confirm the relative quantitative expression levels. Primer pairs used were specifically designed and synthesized by Qiagen GMBH, Valencia, CA. One-step real-time RT-PCR reactions were performed in a 50-µL volume containing a solution of 1× supermix (iQ SYBR Green; Bio-Rad), 1 µM mix of forward and reverse primer, and 5 µL total RNA. A four-step protocol was used: denaturation, 3 minutes at 95°C; amplification and quantification, 40 cycles for 15 seconds at 95°C and for 30 seconds at 60°C; melting curve, 60 to 95°C with a heating rate of 0.5°C per second; followed by cooling (MyiQ Single Color Real-Time PCR Detection System, Bio-Rad). A single-peak melting curve was observed for each gene product. Relative quantitative expression levels were determined for each gene. All results were displayed as an expression ratio normalized against *β*-actin expression levels using the 2^−ΔΔ*C*^ *_T_* method.

### Streptozotocin (STZ)-induced diabetes

Twelve-week-old C57Bl/6 mice were used for this study. Diabetes was induced in the mice by intraperitoneal injection of 170 µg/g of streptozotocin (Sigma Aldrich, St. Louis, MO). Controls were injected with a vehicle (0.01M sodium citrate buffer). On the third day after STZ treatment, mouse tail veins were bled to test the hyperglycemic status using a blood glucose detection kit (Lifescan; Johnson & Johnson, Milpitas, CA). Mice with blood sugar levels of >300 mg/dL on the third day after STZ treatment were considered diabetic. Eye drop treatment containing 1% apoEdp or vehicle PBS was continued 4 times a day for 14 consecutive days. Intra-peritoneal injection containing 40 mg/kg of apoEdp or vehicle control PBS was administered once daily for 14 consecutive days. On the 15^th^ day post-treatment, mice were euthanized, the eyes enucleated, and the retinae were dissected for mRNA or Western blot analysis.

### Corneal penetration assay

Mouse eyes were treated with 1% apoEdp drops. At 0, 1, 2, 3, hours post-treatment, mice were sacrificed and eyes were enucleated. The globes were washed with sterile PBS three times and aqueous humor was aspirated using a 27-gauge needle and 1 mL syringe. The mouse aqueous humor samples were mixed with 1 ml of 0.1% formic acid solution and allowed to stand for 5 minutes. The samples were filtered with a 0.2-micron filter and analyzed using a Waters Acquity UPLC system (Milford, MA) with mass spectrophotometrical detection. Initially, the mass range for the peptide was scanned and several peaks found that corresponded to different charged states of the peptide. These peaks included 483.9 m/z 604.4 m/z, and 805.6 m/z. We used the 484.9 peak for the analysis because it was the strongest. For the UPLC analysis, we used a BEH300 C4 column 2.1 mm×100 mm×1.7 µm. A 2 component mobile phase was used with component 1:0.1% formic Acid, and Component 2: Acetonitrile with 0.1% formic acid. The flow rate was 0.2 ml/min and gradient elution was used with a linear gradient.

### Immunohistochemistry

Following euthanasia, enucleated eyes were immersed in 10% buffered formalin and subsequently embedded in paraffin. Serial sections (6 µ thick) were prepared out of paraffin blocks. Following deparaffinization, sections were hydrated by sequential immersion in xylene and graded alcohol solutions, and were treated with proteinase K for 5 minutes at 37°C. Three randomly chosen fields per eye section were viewed to generate the quantitative immunohistochemical staining results. Sections were processed using an immunohistochemistry kit (Pierce). Slides were incubated overnight at 4°C with anti-ZO-1 (1∶100; Santa Cruz Biotechnology) and donkey anti-rabbit IgG (1∶400, Santa Cruz Biotechnology) secondary antibodies. The slides were mounted in aqueous mounting medium (supplied with kit) and observed by light microscopy (Carl Zeiss Meditec, Chester, VA).

### Statistical analysis

Statistical differences between groups were evaluated with the Student unpaired *t*-test (two-tailed). Mean ± SD is shown. *P*≤0.05 was considered significant.
